# Assessment of Socio‐Demographic and Clinical Characteristics of Stroke Patients of a Tertiary Hospital in Bangladesh: A Cross‐Sectional Study

**DOI:** 10.1002/hsr2.72284

**Published:** 2026-04-05

**Authors:** Sree Shib Shankar Devnath Debu, Ryadul Alam, Israt Jahan Eti, Dipak Kumar Mitra

**Affiliations:** ^1^ Department of Public Health North South University Dhaka Bangladesh; ^2^ Department of Public Health & Informatics Jahangirnagar University Dhaka Bangladesh; ^3^ National Institute of Neurosciences and Hospital Dhaka Bangladesh

**Keywords:** clinical characteristics, diabetes, dyslipidemia, hypertension, socio‐demographic characteristics, stroke type

## Abstract

**Background and Aims:**

Stroke is a major public health concern with significant socio‐economic implications. Understanding the sociodemographic and clinical characteristics of stroke patients is essential for effective prevention, management, and planning of healthcare services. This study aims to assess the socio‐demographic and clinical profiles of stroke patients in a tertiary hospital in Bangladesh.

**Method:**

A cross‐sectional study was conducted among stroke patients admitted to the tertiary hospital. Data on socio‐demographic characteristics, clinical factors, risk factors, and stroke characteristics were collected through structured interviews and medical record reviews. Descriptive statistics were used to analyze the data. A total of 200 participants were taken in this study.

**Results:**

Majority of the respondents were females (54.0%), and significant proportion were above 60 years of age (57.0%). Hypertension (94.0%), diabetes (70.5%), and dyslipidemia (53.5%) were prevalent among the patients. The most common type of stroke observed was hemorrhagic (37.0%). Headaches (71.5%), vomiting (66.5%), and convulsions (61.0%) were reported during stroke episodes. The study also highlighted the high prevalence of speech involvement (59.5%) and right‐side paralysis (36.0%) among the patients. The findings suggest the need for targeted interventions to manage risk factors and improve stroke care. Significant factors of stroke were Diabetes and Dyslipidemia.

**Conclusion:**

This study provides valuable insights into the socio‐demographic and clinical characteristics of stroke patients in a tertiary hospital in Bangladesh. The findings underscore the importance of stroke prevention strategies, including effective management of risk factors such as hypertension, diabetes, and dyslipidemia. The results also highlight the need for comprehensive stroke care services, including rehabilitation, to address the functional impairments associated with stroke.

## 1 Introduction

Stroke is one of the leading non‐communicable diseases (NCDs) globally, contributing significantly to mortality, disability, and healthcare costs [1]. It is the second leading cause of death and a major cause of long‐term disability worldwide, affecting about 10.3 million people, causing 6.5 million deaths, and leaving approximately 26 million survivors with some form of impairment each year [2, 3]. Over the past three decades, the incidence of stroke in low‐ and middle‐income countries (LMICs) has nearly doubled, increasing from 56 to 117 per 100,000 person‐years [2]. Nearly 80% of all NCD‐related deaths occur in LMICs. High blood pressure and tobacco use are the most important modifiable risk factors, while atrial fibrillation, heart failure, and ischemic heart disease also play major roles [2, 4]. Although the incidence of stroke has declined in many developed countries due to better control of hypertension and reduced smoking, the overall number of stroke cases continues to rise globally due to population ageing [4]. In South‐East Asia, stroke remains a growing public health concern. More than half of all NCD‐related deaths in this region are linked to stroke and related diseases [4]. Studies from Bangladesh and neighboring countries show that stroke incidence increases with age and is more common among men. Hypertension, diabetes, and smoking are the most frequently reported risk factors [5, 6].

Globally, studies have highlighted the association between socio‐economic status and stroke incidence. In Sweden lower education and income levels were associated with a higher risk of stroke [5]. Ischemic stroke was more prevalent than hemorrhagic stroke, with differences in age distribution and risk factor profiles between the two subtypes [7]. Understanding these subtype‐specific characteristics is essential for tailoring stroke management strategies and improving patient outcomes [8]. Recent data in Egypt and in the United Arab Emirates point to the threat of hemorrhagic transformation (HT) in patients with atrial fibrillation (AF) who have embolic stroke with alteplase [9]. HT was observed in 28.9% of a group of patients.

In Bangladesh, a recent national population‐based survey involving a representative sample of the population identified a lifetime prevalence rate of 11.39 per 1,000 adults of stroke. The prevalence was significantly greater in persons of age ≥ 60 years and that males were almost twice as likely to possess it than females. In rural region of Bangladesh had found even lower rates, although it still highlighted the increasing burden of cerebrovascular disorders in that population. The economic and health impacts are huge that stroke survivors tend to need long‐term care, experience disability and lower quality of life, and put a significant strain on healthcare systems operating in resource‐constrained environments [2]. Recent national data indicate that stroke accounts for about 16% of deaths among the elderly in Bangladesh, ranking above ischemic heart disease and respiratory infections [2]. Despite this high burden, there is limited comprehensive data on the socio‐demographic and clinical characteristics of stroke patients in the country, and hospital‐based registries remain scarce.

Although this increased incidence of stroke in Bangladesh, there are still a number of gaps in the evidence. The number of available studies is community surveys or small hospital‐based series; few of them have reported a thorough description of stroke patients hospitalized in tertiary care facilities in Bangladesh comprising of socio‐demographic factors (age, sex, socio‐economic status, residence), clinical factors (stroke subtype, severity, comorbidities) and risk factors. These parameters need to be characterized to customize the pathways of acute stroke care to tertiary hospitals and to inform national policy and resource allocation in a health‐system burdened increasingly by non‐communicable disease.

To address this gap, the present study aims to assess the socio‐demographic and clinical characteristics of stroke patients admitted to a tertiary hospital in Bangladesh. The findings will help identify key risk factors and vulnerable groups, providing evidence to support the development of targeted prevention, treatment, and rehabilitation strategies for stroke management in Bangladesh.

## 2 Methodology

### 2.1 Study Design & Study Period

The research took a cross‐sectional approach. Data was collected from Bangabandhu Sheikh Mujib Medical University (BSMMU), a tertiary level hospital in Bangladesh. This study was carried out between December 2024 and March 2025.

### 2.2 Sample Size & Sampling Technique

A total of 200 participants were selected using a convenient sampling method, balancing statistical adequacy with practical feasibility.

### 2.3 Inclusion Criteria

Participants included in this study met the following criteria: i) **Stable medical condition**, absence of any acute life‐threatening illness (e.g., renal failure, hepatic failure, or acute myocardial infarction) or hemodynamic instability at the time of recruitment, ii) **Age** ≥ **18 years** (no upper age limit was applied), iii) **Acceptable cognitive ability** to provide informed consent and participate in assessments, either directly or through a legally authorized representative when necessary and iv) **Capability of independent daily activities**, assessing pre‐stroke independence helps ensure that post‐stroke functional limitations are attributed to the stroke itself rather than pre‐existing disabilities.

### 2.4 Exclusion Criteria

The exclusion criteria for the study were: i) Hemiplegia caused by cancer or trauma, (ii) Pathologic cerebellar abnormalities, and (iii) Significant cognitive impairment.

### 2.5 Data Collection Tools

The study was conducted through a face‐to‐face interview. A questionnaire will contain questions that are open‐ended as well as closed ended. Closed‐ended items will have yes/no or multiple‐choice answers, and the answers will be predetermined in many situations. This questionnaire was developed by reviewing existing literature [10, 11].

### 2.6 Data Collection Procedures

The data was collected using a semi‐structured questionnaire through face‐to‐face interview. Prior permission was obtained from the college authorities to conduct the survey. The participants were provided with a brief explanation of the study's purpose, and I assured them of their anonymity and confidentiality. Data collection was conducted in person, and participants were requested to response to the questions voluntarily. They were requested to give about 30 min to complete the interview. The authors had access to anonymized medical records, ensuring that no personally identifiable information of individual participants was available during or after data collection. Permission to access and use the medical records was obtained from the relevant medical authority of the tertiary hospital.

### 2.7 Study Variables

The study considered a few important aspects that could affect the study's outcome. The independent variables are age, marital status, educational qualification, monthly household income, family type, current residence, Hypertension, Diabetes, Dyslipidemia, smoking habit, physical activity, sleeping time etc. The dependent variable is Type of stroke (Hemorrhagic stroke/Ischemic stroke).

### 2.8 Definition of Dependent Variables

***Ischemic Stroke*** **:** A sudden brain failure caused by blockage of blood supply to the brain, caused by either thrombosis or embolism, which causes cerebral infarction [12].

***Hemorrhagic Stroke*** **:** Bleeding in the brain parenchyma or the subarachnoid space caused by the burst of cerebral blood vessels [13].

### 2.9 Statistical Analysis

The collected data was carefully reviewed, coded, scored, and entered Microsoft Excel. To ensure the quality of the data, missing and duplicate values were checked for errors. Statistical analysis was performed using SPSS version 25. The data that was collected and used to summarize the socio‐demographic, clinical, and risk factor characteristics of the participants were summarized using descriptive statistics. Gender, marital status, education, comorbidities, and type of stroke were categorical variables, where they were presented as frequencies and percentages. The Chi‐square (χ2) test was used to investigate the associations between categorical independent variables and the kind of stroke (hemorrhagic or ischemic). The Fisher's exact test was applied where cell counts were below five. The bivariate analysis displayed to be incorporated in a binary logistic regression model in determining the independent predictor of stroke type.

The dependent variable, type of stroke was coded 1‐hemorrhagic and 0‐ischemic according to the findings of CT scan. The binary logistic regression was employed since the outcome variable was a dichotomous variable with the ability to estimate the adjusted odds ratios (AORs) and 95% confidence intervals (CIs) after the variables had been adjusted to control the possible confounders. Statistical tests were all two‐tailed and *p*‐value of less than 0.05 was taken to be statistically significant.

### 2.10 Ethical Considerations

It received approval from the “Institutional Review Board” at National Institute of Neurosciences and Hospital, Memo No: **IRB/NINS/2025/408**. Prior to data collection, all participants gave their informed written consent. Participation was entirely voluntary, and information was kept completely confidential. All procedures of the present study were conducted in accordance with human involving research guidelines (e.g., Helsinki declaration). Informed consent was obtained from each participant where the study's procedures, objectives, and confidentiality about their information, etc. were clearly documented. The data were collected anonymously and analyzed using numerical codes.

## 3 Result

The sample are females, comprising 54.0%, while males make up 46.0% of the total. The majority of respondents, 57.0%, are above 60 years of age, while 43.0% are up to 60 years old. The highest percentage of respondents, 49.0%, have an education level above secondary. 29.0% have intermediate education, and 22.0% have an honors degree. The largest occupational group among respondents is housewives, accounting for 45.5%. Other significant groups include businessmen (20.5%), employees (12.0%), and retirees (15.5%). The majority of respondents, 91.0%, are married, while the percentages for unmarried and divorced respondents are the same at 4.5%. The distribution of monthly family income is almost equal, with 48.0% having an income up to 30000 BDT and 52.0% having a higher income. The majority of respondents (54.5%) reported their health status as poor, while 45.5% reported it as good. The distribution of hospital reporting times indicates that 51.5% of respondents stayed in the hospital for 3–24 h, 28.0% stayed for more than 24 h, and 20.5% stayed for less than 3 h (Table 1).

**Table 1:** Socio‐demographic characteristics of the respondents.

Variables	*n* (%)
**Gender**	
Male	92 (46.0)
Female	108 (54.0)
**Age**	
Up to 60 years	86 (43.0)
More than 60 years	114 (57.0)
**Education**	
> Secondary	98 (49.0)
Intermediate	58 (29.0)
University	44 (22.0)
**Occupation**	
Student	7 (3.5)
Housewife	91 (45.5)
Employee	24 (12.0)
Businessman	41 (20.5)
Retired	31 (15.5)
Unemployed	6 (3.0)
**Marital status**	
Married	182 (91.0)
Unmarried	9 (4.5)
Divorced	9 (4.5)
**Monthly family income**	
Up to 30000 BDT (Bangladeshi Currency)	96 (48.0)
More than 30000 BDT	104 (52.0)
**Residence**	
Rural	58 (29.0)
Semi‐urban	61 (30.5)
Urban	81 (40.5)
**Self‐reported health status**	
Poor	109 (54.5)
Good	91 (45.5)
**Times of reporting hospital**	
< 3 hrs	41 (20.5)
3–24 hrs	103 (51.5)
> 24 hrs	56 (28.0)

The largest portion of respondents, accounting for 94.0%, indicates that they have hypertension. Most respondents have diabetes, with 70.5% indicating they have this risk factor. 53.5% of the respondents indicate the presence of dyslipidemia as a risk factor. The majority of the respondents do not have a smoking habit, with 68.0% indicating they do not smoke. A significant portion of 49.5% of the respondents do not participate in regular physical activity. Approximately half (50.0%) of the respondents have a sleeping duration of 6–8 h The majority of respondents have a previous history of stroke, with 51.0% indicating they have experienced a stroke in the past (Table 2).

**Table 2:** Probable risk factors of the respondents.

Variables	*n* (%)
** Hypertension**	188 (94.0)
** Diabetes**	141 (70.5)
** Dyslipidemia**	107 (53.5)
** Smoking habit (present)**	64 (32.0)
** Doing physical activity**	101 (50.5)
** Sleeping time**	
> 6 hrs	43 (21.5)
6–8 hrs	100 (50.0)
< 8 hrs	57 (28.5)
** Previous history of stroke**	102 (51.0)

The respondents' CT scan results indicate that the majority of lesions are categorized as hemorrhagic (37.0%), followed by ischemic (28.5%) and ICH (29.5%). Only a small number of cases involve cerebellar lesions (1.5%) or SAH (3.5%). The highest percentage of stroke occurrences was reported at noon (30.0%), while the lowest percentage was observed during the night (21.5%). Most respondents (71.5%) reported experiencing headaches during their stroke episodes, while the remaining 28.5% did not have headaches. A significant portion of respondents (66.5%) experienced vomiting along with their stroke episodes, while 33.5% did not have vomiting symptoms. Approximately 61.0% of the respondents had convulsions during their stroke episodes, while 39.0% did not experience convulsions. In 59.5% of the cases, the respondents' speech was affected during their stroke episodes, while 40.5% did not experience speech involvement. Most respondents did not exhibit hemiplegia or monoplegia (41.0%), while 36.0% experienced right‐side paralysis and 18.0% had left‐side paralysis. A small portion (5.0%) reported bilateral involvement. During their stroke episodes, 57.0% of the respondents were unconscious, while the remaining 43.0% remained conscious (Table 3).

**Table 3:** Clinical characteristics of the respondents.

Variables	*n* (%)
** Pattern of lesion infraction in CT scan**	
Ischemic	57 (28.5)
Hemorrhagic	74 (37.0)
ICH	59 (29.5)
Cerebellar	3 (1.5)
SAH	7 (3.5)
** Times of occurrence**	
Morning	53 (26.5)
Noon	60 (30.0)
Afternoon	44 (22.0)
Night	43 (21.5)
** Headache**	143 (71.5)
** Vomiting**	133 (66.5)
** Convulsion**	122 (61.0)
** Speech involvement**	119 (59.5)
** Hemiplegia/monoplegia (present)**	
Right	72 (36.0)
Left	36 (18.0)
Bilateral	10 (5.0)
No	82 (41.0)
** Unconscious**	114 (57.0)

Abbreviations: CT, computed tomography; ICH, intracerebral hemorrhage; SAH, subarachnoid hemorrhage.

There is no statistically significant association between gender, age, education, occupation, marital status, monthly family income, residence, self‐reported health status, times of reporting hospital, and the type of stroke (hemorrhagic or ischemic) (Table 4). There are statistically significant associations between diabetes and dyslipidemia (*p*‐value < 0.05) with the type of stroke. Respondents with diabetes have a higher prevalence of hemorrhagic stroke, while those with dyslipidemia have a higher prevalence of ischemic stroke. Hypertension, smoking habits, physical activity, sleeping time, and a previous history of stroke show no significant associations with the type of stroke (Table 5). Also, there are significant associations between the pattern of lesion infraction in CT scan and the type of stroke (*p*‐value < 0.05). Hemorrhagic stroke is more prevalent in cases of CT scan showing hemorrhagic lesions, ICH, cerebellar involvement, and SAH. However, no significant associations were found between the type of stroke and times of occurrence, headache present, vomiting present, convulsion present, speech involvement present, hemiplegia/monoplegia present, or unconscious present (Table 6).

**Table 4:** Association of socio‐demographic characteristics with type of stroke.

Variables	Category	Type of stroke		*χ* ^2^	*p*‐value
		Hemorrhagic stroke (%)	Ischemic stroke (%)		
**Gender**	Male	56.5%	43.5%	0.433	0.511
	Female	61.1%	38.9%		
**Age**	Up to 60 years	66.3%	33.7%	3.305	0.069
	More than 60 years	53.5%	46.5%		
**Education**	> Secondary	67.3%	32.7%	5.565	0.062
	Intermediate	51.7%	48.3%		
	University	50.0%	50.0%		
**Occupation**	Student	71.4%	28.6%	6.359*Fisher exact test	0.270
	Housewife	65.9%	34.1%		
	Employee	41.7%	58.3%		
	Businessman	51.2%	48.8%		
	Retired	58.1%	41.9%		
	Unemployed	66.7%	33.3%		
**Marital status**	Married	59.9%	40.1%	1.022*	0.741
	Unmarried	55.6%	44.4%		
	Divorced	44.4%	55.6%		
**Monthly family income**	Up to 30000BDT	65.6%	34.4%	3.350	0.067
	More than 30000 BDT	52.9%	47.1%		
**Residence**	Rural	55.2%	44.8%	4.852	0.088
	Semi‐urban	70.5%	29.5%		
	Urban	53.1%	46.9%		
**Self‐reported health status**	Poor	63.6%	36.4%	3.350	0.067
	Good	53.3%	46.7%		
**Times of reporting hospital**	< 3 hrs	53.7%	46.3%	2.608	0.271
	3–24 hrs	56.3%	43.7%		
	> 24 hrs	67.9%	32.1%		

* Fisher exact test.

**Table 5:** Association of probable risk factors with type of stroke.

Variables	Category	Type of stroke		*χ* ^2^	*p*‐value
		Hemorrhagic stroke (%)	Ischemic stroke (%)		
**Hypertension**	Yes	58.5%	41.5%	0.310	0.578
	No	66.7%	33.3%		
**Diabetes**	Yes	51.8%	48.2%	10.320	**0.001** **
	No	76.3%	23.7%		
**Dyslipidemia**	Yes	48.6%	51.4%	10.292	**0.001** **
	No	71.0%	29.0%		
**Smoking habit**	Yes	59.4%	40.6%	0.005	0.941
	No	58.8%	41.2%		
**Physical activity**	Yes	59.4%	40.6%	0.014	0.906
	No	58.6%	41.4%		
**Sleeping time**	> 6 hrs	69.8%	30.2%	4.401	0.111
	6–8 hrs	60.0%	40.0%		
	< 8 hrs	49.1%	50.9%		
**Previous history of stroke**	Yes	52.9%	47.1%	3.159	0.076
	No	65.3%	34.7%		

*Note:* Bold values indicate statistically significant result.

** *p* < 0.001.

**Table 6:** Association of clinical characteristics with type of stroke.

Variables	Category	Type of stroke		Chi square	*p*‐value
		Hemorrhagic stroke (%)	Ischemic stroke (%)		
**Pattern of Lesion Infraction in CT scan**	Ischemic	28.1%	71.9%	35.593*	0.001**
	Hemorrhagic	73.0%	27.0%		
	ICH	64.4%	35.6%		
	Cerebellar	100.0%			
	SAH	100.0%			
**Times of occurrence**	Morning	56.6%	43.4%	0.888	0.828
	Noon	58.3%	41.7%		
	Afternoon	56.8%	43.2%		
	Night	65.1%	34.9%		
**Headache present**	Yes	56.6%	43.4%	1.152	0.283
	No	64.9%	35.1%		
**Vomiting present**	Yes	55.6%	44.4%	1.854	0.173
	No	65.7%	34.3%		
**Convulsion present**	Yes	55.7%	44.3%	1.376	0.241
	No	64.1%	35.9%		
**Speech involvement present**	Yes	57.1%	42.9%	0.419	0.517
	No	61.7%	38.3%		
**Hemiplegia/monoplegia present**	Right	61.1%	38.9%	1.979	0.577
	Left	66.7%	33.3%		
	Bilateral	60.0%	40.0%		
	No	53.7%	46.3%		
**Unconscious present**	Yes	55.3%	44.7%	1.530	0.216
	No	64.0%	36.0%		

Abbreviations: CT, computed tomography; ICH, intracerebral hemorrhage; SAH, subarachnoid hemorrhage.

* Fisher exact test.

** *p* < 0.001.

**Figure 1:**
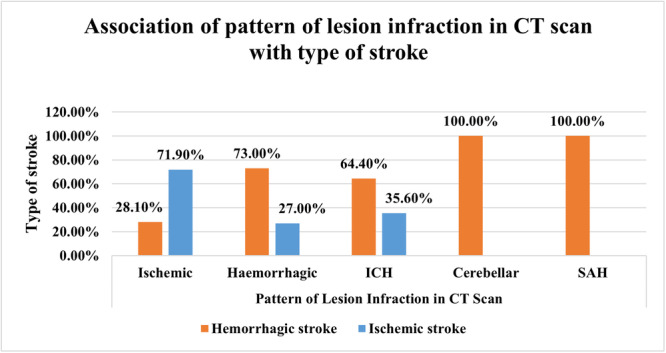
Pattern of lesion infraction in CT scan with stroke type.

Figure 1 illustrates that the majority of cases with the pattern of lesion infarction in CT scans were associated with hemorrhagic strokes (73.0%), while a smaller proportion was linked to ischemic strokes (28.1%). Among hemorrhagic strokes, a significant portion was categorized as ICH (64.4%). Additionally, all cases within the pattern of lesion infarction in CT scans were associated with cerebellar strokes and SAH.

The result of the binary logistic regression analysis. Only two variables were found significant in the adjusted model. Diabetic participants were nearly three times more likely to report Hemorrhagic stroke compared to the non‐diabetic group (AOR = 2.99, 95% CI = 1.510‐5.938, *p* = 0.027). Participants who had Dyslipidemia were three times more likely to report Hemorrhagic stroke compared to non‐Dyslipidemia participants (AOR = 2.88, 95% CI = 1.098‐4.779, *p* = 0.012) (Table 7).

**Table 7:** Binary logistic regression of the variables.

Variables	Unadjusted model		Adjusted model^a^	
	COR (95% CI)	*p*‐value	AOR (95% CI)	*p*‐value
**Age**				
Up to 60 years	0.586 (0.328–1.045)	0.070		
More than 60 years	Reference			
**Gender**				
Male	0.827 (0.470–1.456)	0.511		
Female	Reference			
**Education**				
> Secondary	0.485 (0.235–1.002)	0.051		
Intermediate	0.933 (0.426–2.044)	0.863		
University	Reference			
**Occupation**				
Student	0.800 (0.067–8.474)	0.853		
Housewife	1.033 (0.179–5.958)	0.971		
Employee	2.800 (0.427–18.375)	0.283		
Businessman	1.905 (0.313–11.575)	0.484		
Retired	1.444 (0.229–9.106)	0.853		
Unemployed	Reference			
**Marital status**				
Married	0.536 (0.139–2.062)	0.364		
Unmarried	0.640 (0.100–4.109)	0.638		
Divorced	Reference			
**Family monthly income**				
Up to 30000BDT	0.588 (0.332–1.040)	0.068		
More than 30000 BDT	Reference			
**Residence**				
Rural	0.919 (0.467–1.809)	0.808	0.861 (0.416–1.782)	0.808
Semi‐urban	0.474 (0.235–0.956)	**0.037** *	0.481 (0.229–1.011)	0.054
Urban	Reference		Reference	
**Diabetes**				
Yes	2.994 (1.510–5.938)	**0.002** **	2.994 (1.510–5.938)	**0.027** *
No	Reference		Reference	
**Dyslipidemia**				
Yes	2.585 (1.438–4.649)	**0.002** **	2.885 (1.098–4.779)	**0.012** *
No	Reference		Reference	
**Sleeping time**				
> 6 hrs	0.418 (0.182–0.962)	**0.040** *	0.418 (0.182–0.962)	0.058
6–8 hrs	0.644 (0.334–1.240)	0.188	0.644 (0.334–1.240)	0.497
< 8 hrs	Reference		Reference	

*Note:* Bold values indicate statistically significant result.

Abbreviations: AOR, adjusted odds ratio; BDT, Bangladeshi Taka; COR, crude odds ratio; CI, confidence interval.

^a^ Adjusted for residence, diabetes, dyslipidemia, and sleeping time.

* *p* < 0.05;

** *p* < 0.01.

## 4 Discussion

This paper examined socio‐demographic and clinical characteristics of stroke patients in a tertiary hospital in Bangladesh. The results suggest that both men and women are affected by stroke, and most of the patients are above 60 years. Age, residence and comorbidities were found to be powerful factors that relate to stroke presentation. The most prevalent risk factors were found to be hypertension, diabetes and dyslipidemia whereas the clinical phenotypes such as lesion patterns on CT scans and the correlating neurological symptoms varied in the case of both ischemic and hemorrhagic strokes.

There were significant correlations between the pattern of the lesion in CT scan and the type of stroke. Hemorrhagic strokes were more common among the hemorrhagic lesions, ICH, cerebellar lesions, and SAH. Though there was a higher level of females in the sample, it does not mean that there are a greater number of women experiencing stroke in general because it can be age related based on the demographics and the increased life expectancy of women. However, such outcomes indicate the need to pay attention to risk profiles peculiar to gender and possible differences in stroke presentation and treatment.

Age‐specific assessment and care plans underline the necessity to pay special attention to stroke as a condition that mostly impacts the elderly ages as its prevalence is among people over 60 years of age [11]. Education background also did not emerge to be a protective factor with a larger percentage of the patients having education of above secondary level indicating that stroke was not different in patients at varying levels of education [14]. Occupational distribution indicating that housewives are the most numerous group is indicative of the fact that stroke affects people in their productive age, taking away the daily routine and caregiving duties [15].

Clinically, diabetes showed significant morbidity with hemorrhagic stroke, and dyslipidemia with ischemic stroke, which is additional support of the value of controlling the conditions to reduce subtype‐specific risks [16]. These findings align with previous researches indicating that diabetes is associated with a higher incidence and greater morbidity of stroke, particularly among patients with type 1 diabetes or poor glycemic control [17, 18]. Similarly, elevated total cholesterol, LDL, and triglycerides, along with low HDL, have been consistently linked to increased risk and worse prognosis in ischemic stroke patients [19, 20].

The temporal trends in the incidence of stroke showed that it was more common at noon, in contrast researchers finds a clear circadian rhythm in stroke occurrence [21], with the highest risk and frequency of onset in the morning hours, particularly between 6 AM and noon. The heterogeneity of the clinical appearance of stroke was manifested by neurological symptoms, which included headache, vomiting, convulsions, speech involvement, and paralysis [22].

In middle east patients treated with alteplase showed higher baseline NIHSS scores related with admission hyperglycemia, and post‐alteplase intracerebral hemorrhage independently [23]. This aligns with our findings that diabetes and dyslipidemia are key contributors to stroke severity and highlights the importance of careful monitoring and management of these risk factors to improve post‐stroke outcomes.

In general, the results are consistent with the available sources of literature on Bangladesh and other South Asian communities, indicating that the characteristics of stroke are affected by socio‐demographic factors and comorbidities [24, 25]. The results may be put into perspective in comparison with other studies within the countries and various health care settings to aid in the formulation of specific preventive and clinical plans [26, 27]. The patterns of the CT scan lesions and stroke subtypes have been found to be associated with the existing stroke pathology, which indicates the importance of the imaging tool in proper diagnosis and classification [28].

This research paper has several limitations as well. It was carried out in one tertiary hospital, which restricts the generalizability of results. There is a possibility of missing the diversity of the stroke patients in Bangladesh due to the sample size. Data collected on the self are prone to the issue of recall bias, and the cross‐sectional design does not allow causal conclusion. Such significant variables as wider socioeconomic variables, cultural factors, and access to healthcare have not been evaluated. There might also be selection bias since it was only the patients seeking care in this hospital that were used. Multicenter and longitudinal research in the future is justified to offer a more detailed description of stroke manifestations in the Bangladeshi community [29].

## 5 Conclusion

This study highlights that stroke patients in a tertiary hospital in Bangladesh are predominantly older adults, with hypertension, diabetes, and dyslipidemia identified as the most common comorbid risk factors. Distinct patterns were observed between stroke subtypes and CT scan lesion characteristics, along with varied neurological manifestations. These findings emphasize the importance of early detection and effective management of modifiable risk factors, particularly among the elderly population. Strengthening preventive strategies and improving clinical management of cardiovascular comorbidities may help reduce the burden of stroke. Further multicenter and longitudinal studies are recommended to better understand stroke patterns and support the development of targeted prevention and treatment strategies in Bangladesh.

## Author Contributions

**Sree Shib Shankar Devnath Debu:** conceptualization, supervision, methodology, writing – review and editing. **Ryadul Alam:** conceptualization, data curation, formal analysis, writing – original draft, validation. **Israt Jahan Eti:** investigation, data curation, validation, writing – review and editing. **Dipak Kumar Mitra:** conceptualization, supervision, writing – review and editing.

## Funding

The authors have nothing to report.

## Ethics Statement

The study protocol was evaluated and approved by National Institute of Neurosciences and Hospital, Memo No: **IRB/NINS/2025/408**.

## Conflicts of Interest

The authors declare no conflicts of interest.

## Transparency Statement

The lead author Ryadul Alam affirms that this manuscript is an honest, accurate, and transparent account of the study being reported; that no important aspects of the study have been omitted; and that any discrepancies from the study as planned (and, if relevant, registered) have been explained.

## Data Availability

The data and materials supporting the findings of this study are available upon reasonable request. Interested researchers can contact the corresponding author via email to access these resources.
